# Managing Cytomegalovirus Infection in Lung Transplant Recipients in Real Life: Results of a French Multicenter Survey

**DOI:** 10.3389/ti.2025.15224

**Published:** 2025-10-29

**Authors:** Tiphaine Goletto, Kinan El Husseini, Antoine Roux, Mathilde Briard, Gaelle Dauriat, Benjamin Renaud-Picard, Claire Merveilleux du Vignaux, Loic Falque, Benjamin Coiffard, Thomas Villeneuve, Xavier Demant, Adrien Tissot, Domitille Mouren, Francois M. Carlier, Sophie Alain, Jonathan Messika, Vincent Bunel

**Affiliations:** ^1^ Service de Pneumologie B et Transplantation Pulmonaire, Hôpital Bichat-Claude Bernard, APHP.Nord-Université Paris Cité, Paris, France; ^2^ Institut National de la Santé et de la Recherche Médicale (INSERM), Centre de Recherche de l’inflammation, Université Paris Cité, Paris, France; ^3^ Service de Pneumologie, Hôpital Foch, Suresnes, France; ^4^ Service de Pharmacie, Hôpital Bichat-Claude Bernard, APHP.Nord-Université Paris Cité, Paris, France; ^5^ Service de Pneumologie, Hôpital Marie Lannelongue, Le Plessis Robinson, France; ^6^ Groupe de Transplantation Pulmonaire des Hôpitaux Universitaires de Strasbourg, Inserm-Université de Strasbourg, Strasbourg, France; ^7^ Service de Pneumologie, Hospices Civils de Lyon, Groupement Hospitalier Est (GHE), Institut National de la Santé et de la Recherche Médicale (INSERM), Lyon, France; ^8^ Service Hospitalier Universitaire Pneumologie et Physiologie, Pôle Thorax et Vaisseaux, Centre Hospitalo-Universitaire Grenoble Alpes, Grenoble, France; ^9^ Service de Pneumologie et Equipe de Transplantation Pulmonaire, Centre Hospitalo-Universitaire de Marseille, Assistance-Publique Hôpitaux de Marseille, Hôpital Nord, Marseille, France; ^10^ Aix-Marseille Université, Marseille, France; ^11^ Centre Hospitalo-Universitaire de Toulouse, Hôpital Larrey, Toulouse, France; ^12^ Service de Pneumologie, Hôpital Haut-Lévêque, Centre Hospitalo-Universitaire de Bordeaux, Bordeaux, France; ^13^ Service de Pneumologie, Institut du thorax, Centre Hospitalo-Universitaire Nantes, Nantes, France; ^14^ Centre de Transplantation Pulmonaire, Centre Hospitalo-Universitaire UCL Namur, Yvoir, Belgium; ^15^ Laboratoire de Virologie, Centre National de Référence Herpèsvirus, Limoges, France

**Keywords:** cytomegalovirus, lung transplant, questionnaire, practices, guidelines

Dear Editors, Cytomegalovirus (CMV) infection remains a major cause of morbidity and mortality following lung transplantation (LTx), with lung recipients facing particularly high risk due to substantial lung-associated lymphoid tissue harbouring latent CMV [1]. Beyond direct effects, CMV infection increases risks for acute rejection, chronic allograft dysfunction, and opportunistic infections. While international guidelines provide recommendations for CMV management [2–4], real-world adherence in LTx centres remains poorly characterized, particularly given that they represented only 15% of transplant centres in recent broader surveys despite bearing the highest CMV burden [5].

We conducted a cross-sectional survey of 10 French-speaking LTx centres [9 out of 11 French centres (82%) and 1 out of 4 Belgian centres (25%)] between September 2022 and February 2023, using a comprehensive questionnaire addressing CMV prevention, diagnosis, treatment, and resistance management. Fifteen physicians participated, with 13 of 15 (86%) reporting adherence to centre-specific protocols that varied between institutions. All physicians surveyed were pulmonologists and lung transplant specialists, who routinely manage LTx patients and CMV infection in this population. Details regarding our methodology, the questionnaire in itself, as well as the full responses, are available in our Supplementary Material.

Our findings revealed substantial heterogeneity in CMV management practices with significant deviations from established guidelines (Figure 1). Most strikingly, prophylaxis duration showed concerning variability: in seropositive recipients (R+), 5 of 15 respondents (33%) used only 3 months of prophylaxis despite guidelines recommending 6–12 months [3, 4], while 9 of 15 (60%) used 6 months and 1 of 15 (7%) used 12 months. For high-risk donor-positive/recipient-negative (D+/R-) patients, 11 of 15 (73%) appropriately used 12-month prophylaxis, though 4 of 15 (27%) used shorter durations. In R+ patients with short telomere syndrome, which is associated with impaired CMV immunity and increased treatment toxicity [6], 10 of 13 respondents (84%) used standard valganciclovir prophylaxis, with 2 of 13 (16%) employing alternative approaches such as anti-CMV immunoglobulins or valaciclovir.

**FIGURE 1:**
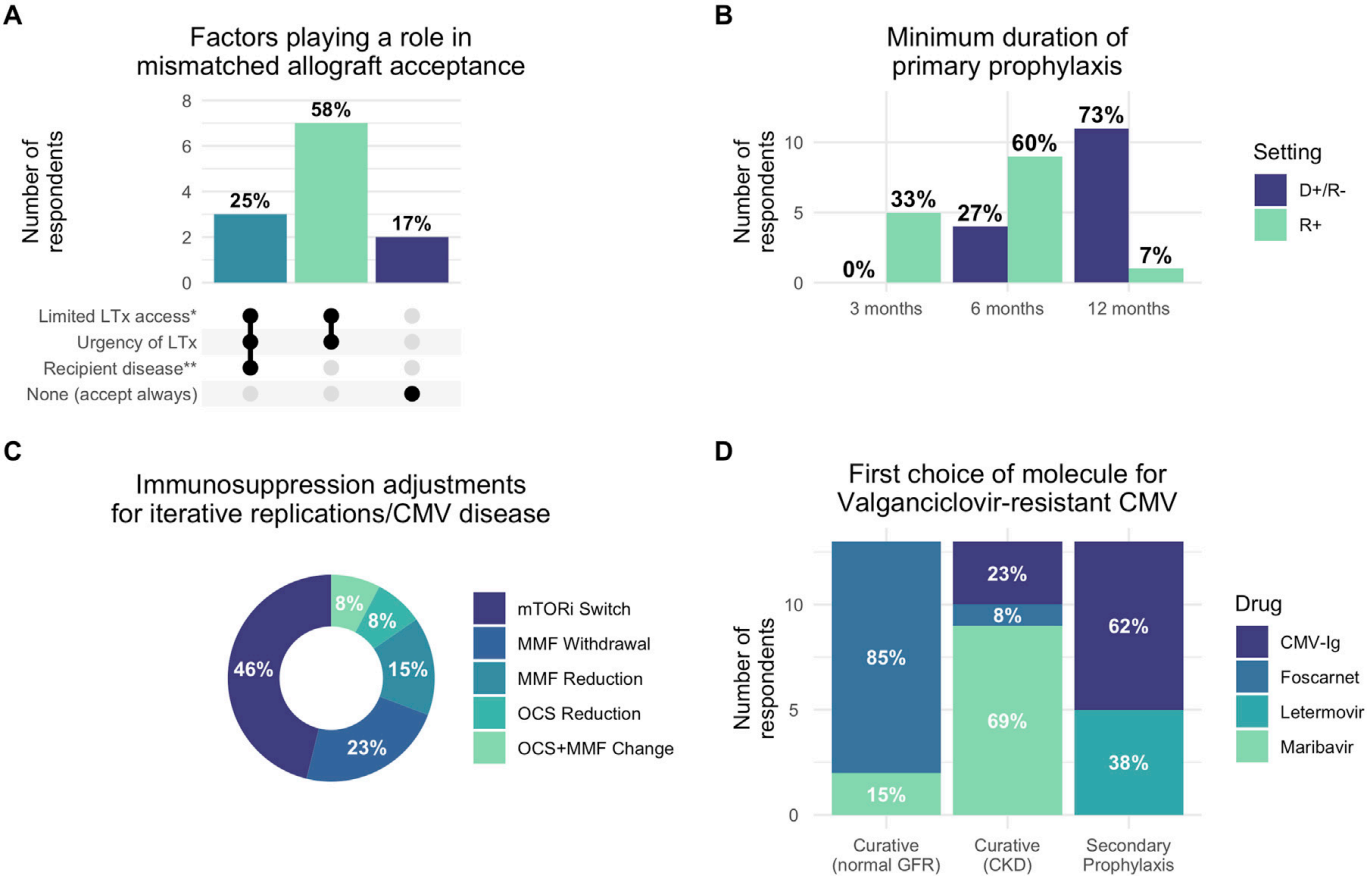
Reported clinical practices for CMV management in lung transplantation. **(A)** Factors influencing acceptance of CMV-mismatched allografts based on responses. Dark dots indicate factors considered by each group. **(B)** Minimum duration of primary CMV prophylaxis by donor/recipient serostatus (D+/R- vs. R+). **(C)** Immunosuppression adjustment strategies preferred for recurrent CMV replication or disease. **(D)** First-choice antiviral therapies for valganciclovir-resistant CMV across different treatment contexts (curative treatment for patients with normal glomerular filtration rate or patients with chronic kidney disease, and secondary prophylaxis). Percentages indicate proportion of responses selecting each factor. *Limited LTx access: recipient factors anticipated to limit access to compatible allografts, such as hyperimmunization, rare ABO group or extreme height, favored mismatched allograft acceptance; **Recipient disease: respondents cited mainly short-telomere syndrome-associated pulmonary fibrosis or systemic sclerosis as situations precluding mismatched allograft acceptance. Abbreviations: D+/R-: donor-positive recipient-negative serostatus; R+: recipient-positive serostatus; CMV: cytomegalovirus; LTx: lung transplantation; CKD: chronic kidney disease; GFR: glomerular filtration rate mTORi: mTOR inhibitor; MMF: mycophenolate mofetil; OCS: oral corticosteroids; CMV-Ig: CMV-specific hyperimmune globulin.

Secondary prophylaxis practices diverged markedly from 2018 guidelines that recommended against routine use [3]. After CMV reactivation, 5 of 14 respondents (36%) systematically initiated secondary prophylaxis with an additional 2 of 14 (14%) using it conditionally. Following CMV disease, these proportions increased to 8 of 14 (57%) and 3 of 14 (21%), respectively. All respondents maintained secondary prophylaxis for 3 months. For patients with iterative replications, 11 of 14 (79%) used long-term prophylaxis with durations varying from 3 to 12 months. This widespread adoption likely reflects the clinical reality that LTx recipients experience higher CMV recurrence rates compared to other solid organ transplant recipients.

Post-prophylaxis monitoring also showed substantial variation, with 6 of 15 respondents (40%) performing monthly monitoring in R+ patients, while in D+/R- patients, 5 of 15 (33%) performed monthly monitoring and 4 of 15 (27%) performed weekly monitoring. This heterogeneity emerged despite 2018 guidelines not supporting surveillance after prophylaxis, though updated 2025 guidelines now suggest monitoring in high-risk patients [4]. CMV-specific cellular immune response testing was used by only 4 of 13 respondents (31%), reflecting limited adoption of these newer diagnostic tools despite their potential for personalized management.

Immunosuppression modification was considered by 5 of 13 respondents (38%) for CMV disease and 12 of 13 (92%) for recurrent infections, most commonly involving mTOR inhibitor introduction or antimetabolite reduction. For hematologic toxicity, 10 of 14 (71%) appropriately used hematologic support, though 2 of 14 (14%) modified immunosuppression and 1 of 14 (7%) reduced valganciclovir doses as first-line interventions, potentially increasing resistance risk [7].

Resistant CMV management revealed evolving practices influenced by new therapeutic options, highlighting both opportunities and challenges in this complex clinical scenario. For patients with normal renal function, 11 of 13 (85%) preferred foscarnet over maribavir (2 of 13, 15%), while in renal impairment, maribavir was preferred by 9 of 13 (69%). Anti-CMV immunoglobulins were used by 8 of 12 respondents (67%) for secondary prophylaxis in resistant cases, with letermovir usage varying widely (8 of 13 (61%) never used it, while others employed it in specific scenarios).

The availability of maribavir through compassionate use programs during our survey period and its subsequent broader approval likely influenced these preferences [8]. Nearly all respondents would test for ganciclovir resistance in case of reactivation despite preventive treatment (11 of 13, 85%) or failure of curative treatment (12 of 13, 93%). These findings underscore the challenges clinicians face when managing resistant CMV, particularly the need to balance efficacy against drug-specific toxicity profiles in an already immunocompromised population with limited access to resistance testing.

The widespread practice variation we observed is particularly significant given that participating centres employ similar immunosuppression protocols and serve comparable populations. Our sample comprised nearly all French LTx centres, suggesting these findings reflect national practice patterns. Similar variability has been reported in Italian programmes [9] and broader European surveys [10], indicating these challenges transcend national boundaries.

The clinical implications are concerning. Santos et al. demonstrated that delayed-onset CMV disease following prophylaxis discontinuation occurs in up to 14% of LTx recipients with associated mortality risk [2]. Our finding that one-third of respondents use only 3-month prophylaxis in R+ patients may have significant clinical consequences, particularly when considering that breakthrough infections may increase resistance risk, impacting long-term allograft survival. Encouragingly, many practice variations we documented have been partially addressed in updated 2025 guidelines [4], which incorporate more aggressive secondary prevention strategies and suggest post-prophylaxis monitoring in high-risk patients, reflecting growing recognition of LTx-specific challenges.

While our study has limitations, including modest sample size and focus on French-speaking centres, our comprehensive coverage of French centres provides valuable insights into an underrepresented but high-risk population. The documented practice heterogeneity, particularly deviations from evidence-based recommendations, highlights critical gaps in CMV management standardization. The fact that 86% of respondents follow centre-specific protocols suggests local guidelines themselves diverge from international recommendations. The higher CMV burden in LTx recipients compared to other solid organ transplant populations necessitates specialized management approaches addressing unique challenges including optimal prophylaxis duration and management of patients with conditions like short telomere syndrome. These findings underscore the need for enhanced education, practice standardization initiatives, and generation of LTx-specific evidence to support future guideline development.

In conclusion, this survey reveals significant heterogeneity in CMV management among French-speaking LTx centres, with notable deviations from international guidelines. Given CMV’s substantial impact on LTx outcomes, addressing these variations through enhanced education, standardized protocols, and LTx-specific evidence generation should be a priority for the transplant community.

## Data Availability

The raw data supporting the conclusions of this article will be made available by the authors, without undue reservation.
