# Supplementary material for: Managing Cytomegalovirus Infection in Lung Transplant Recipients in Real Life: Results of a French Multicenter Survey
Source: Transpl Int. 2025 Oct 29;38:15224. doi: 10.3389/ti.2025.15224 (PMC12605176; doi:10.3389/ti.2025.15224)
Supplement: Supplementary file 1 [file DataSheet1.docx]

**Managing Cytomegalovirus Infection in Lung Transplant Recipients in Real Life: Results of a French Multicenter Survey**

**Supplementary Materials**

**Questionnaire design**

The study questionnaire was developed by two transplant pulmonologists (TG and VB), based on a previously published questionnaire,^1^ updated with questions regarding more recent treatment options and about specificities of LTx. The questionnaire was reviewed by two others pulmonologist of different LTx centers (AR and JM). It contained fifty-nine questions about the management of CMV following LTx, including structured (multiple-choice) and open-ended questions. The questions covered practices about CMV infection and prophylaxis management after LTx and included a specific section on patients with short telomere syndrome-related interstitial lung disease.

The questionnaire also included consensual definitions for CMV infection (viral replication, regardless of symptoms), CMV disease (CMV infection associated with organ impairment), preventive strategy (universal prophylaxis over a given period), pre-emptive strategy (systematic PCR monitoring and treatment in the event of infection), 'R+' (positive CMV serology in the recipient), 'D+/R-', also known as 'CMV mismatch' (CMV seropositive donor and the seronegative recipient).

The survey was hosted on cloud-based software (SurveyMonkey®, San Mateo, CA, United States), and a translated version is available below.

1. Grossi PA, Kamar N, Saliba F, et al. Cytomegalovirus Management in Solid Organ Transplant Recipients: A Pre-COVID-19 Survey From the Working Group of the European Society for Organ Transplantation. *Transpl Int*. 2022;35:10332. doi:10.3389/ti.2022.10332

**Questionnaire (English-translated version)**

1/ Center name

2/ How long have you worked in a lung transplant department?

3/ How many transplants are performed per year in your center (on average over the last three years 2019-2020-2021)

4/ What immunosuppression protocol do you use as first-line treatment?

corticoids

tacrolimus

cyclosporine

cellcept

m Tor inhibitor

other (specify)

5/ Do you use induction?

yes

no

6/ If yes, which and in what percentage

7/ Do you use a service protocol for CMV management?

yes

no

8/ What factors influence your protocol?

donor-recipient status

use of induction

other (specify)

9/ What detection method do you use?

Whole blood PCR

PCR on plasma

PCR on serum

Other (specify)

10/ What detection level do you consider significant for starting treatment as a preemptive strategy?

11/ Do you follow up with the PCR in town?

yes

no

12/ What management strategy do you use after transplantation?

Systematically pre-emptive

Systematically preventive

depending on the situation

13/ If your chosen strategy (preventive versus preemptive) differs according to the situation, what factors influence your choice?

D+/R-

in the event of indiction

other (specify)

14/ In the case of a preventive strategy, what are the monitoring procedures under treatment: at what rhythm do you monitor the PCR?

Weekly

/ 15j

Monthly

No monitoring

Other (specify)

15/ In the case of a preemptive strategy, what are the monitoring procedures under treatment: at what rhythm do you monitor the PCR?

Weekly

/ 15j

Monthly

No monitoring

Other

16/ For R+ patients, what preventive treatment do you use (with normal renal function)?

Valganciclovir 900mg/d

Valganciclovir 450mg/d

Anti-CMV immunoglobulin

Other : (specify)

17// For R+ patients, what duration of preventive treatment do you use?

3 months

6 months

12 months

Other (specify)

18/ For D+/R- patients, what preventive treatment do you use (with normal renal function)?

Valganciclovir 900mg/d

Valganciclovir 450mg/d

Anti-CMV immunoglobulin

Other : (specify)

19/ For D+/R- patients, what duration of preventive treatment do you use?

3 months

6 months

12 months

Other (specify)

20/ In case of preventive strategy, do you initiate CMV-targeted therapy in D-/R- patients?

no never

yes always

yes sometimes (specify)

21/ When you stop prophylaxis, do you use a preemptive strategy?

yes, with a well-defined monitoring schedule (service protocol)

yes, without a well-defined monitoring schedule

no

22/ For R+ patients, what PCR monitoring schedule do you use on discontinuation of prophylaxis?

Weekly

Every 15 days

Monthly

Other (specify)

23/ For D+/R- patients, what PCR monitoring schedule do you use on discontinuation of prophylaxis?

Weekly

Every 15 days

Monthly

Other (specify)

24/ How long do you monitor after stopping treatment?

For R+ patients

For D+/R- patients

25/ If yes (question 21), in what situation?

systematically on discontinuation of preventive treatment

systematically on discontinuation of curative treatment

in case of iterative reactivation

other (specify)

26/ At the first asymptomatic reactivation (without organ involvement) in a patient without malabsorption, what treatment do you use?

IV ganciclovir followed by valganciclovir

valganciclovir per os

foscavir IV

foscavir IV followed by valganciclovir per os

Anti-CMV immunoglobulin IV alone

combination

other

27/ If you use a combination, which one?

28/ How long does curative treatment last?

until 2 negative PCRs are obtained one week apart

other (specify)

29/ Do you start prophylaxis after a first reactivation?

yes always

no never

yes according to certain criteria (please specify)

30/ In the case of secondary prophylaxis after a first reactivation, which molecule do you use?

Valganciclovir

anti-CMV immunoglobulin

other ((specify)

31 / For what duration

3 months

6 months

12 months

other duration (specify)

32/ During the management of CMV disease, which molecule do you use?

IV ganciclovir followed by valganciclovir

valganciclovir per os

foscavir IV

foscavir IV followed by valganciclovir per os

Anti-CMV immunoglobulin IV alone

combination

other

33/ How long does curative treatment last?

until 2 negative PCRs are obtained one week apart

other (specify)

34/ Do you initiate secondary prophylaxis in the aftermath?

yes always

no never

yes according to certain criteria (please specify)

35/ If so, which one as a first-line treatment?

valganciclovir

anti-CMV immunoglobulin

other (please specify)

36/ For how long (seondary prophylaxis after CMV disease)

1 month

3 months

6 months

12 months

other duration (specify)

37/ In case of iterative replications, do you envisage a long-term prophylactic treatment?

yes

no

38/ If yes, which one as first-line treatment?

valganciclovir

anti-CMV immunoglobulin alone

letermovir

other (please specify)

39/ For what duration

1 month

3 months

6 months

12 months

Other (specify)

40/ In which situations would you consider modifying immunosuppression?

CMV disease

in case of iterative replications

other (please specify)

41/ In the absence of a significant immunological event (recent acute rejection or alloiimmunization/DSA), what changes in treatment would you consider as first-line treatment in these situations?

discontinuation of anticalcineurins

dosage reduction of anticalcineurins

reduction of corticosteroid dosage alone

discontinuation of cellcept

cellcept dosage reduction alone

introduction of an mTOR inhibitor as a back-up to cellcept, reducing residual anticalcineurin targets

other

42/ After how many episodes of reactivation/infection do you consider replications to be iterative?

43/ In your current practice, do you use an immunological test for CMV?

non

quantiferon CLV

Elispot CMV

other

44/ When you use an immunological test for CMV, what are the procedures involved?

available locally at my center

send a sample to the CNR (Limoges)

other

45/ In which situations do you use an immunological test (Quantiferon CMV or other)?

guiding the discontinuation or maintenance of primary prophylaxis

guide discontinuation or maintenance of secondary prophylaxis

guide strategy in case of iterative replications

other situation

46/ In what situations do you perform resistance genotyping?

never

reactivation despite preventive treatment

when there is no response to curative treatment

other

47/ How many cases of resistance have you identified in the last three years?

48/ In which situations do you test for ganciclovir?

never

systematically

in case of resistance

other situations

49/ What is your first-line approach in the event of poor hematological tolerance of valganciclovir?

hematological support (G CSF or transfusion)

switch antiviral molecule

reduction in valganciclovir dosage

other

50/ If a switch of antiviral molecule is necessary, please specify your first choice as a replacement for prophylactic valganciclovir

51/ What would be your first choice of molecule in the case of resistant CMV (mutation identified) regardless of availability / ATU problems?

With normal renal function

foscavir

maribavir

anti-CMV immunoglobulin

other or combination therapy

52/ What would be your first choice of molecule in the case of resistant CMV (mutation identified), regardless of availability / ATU problems?

In case of renal failure

foscavir

maribavir

anti-CMV immunoglobulin

other or combination therapy

53/ What is your attitude in the case of resistant CMV (mutation identified)?

For secondary prevention

Anti-CMV immunoglobulin

letermovir

foscavir 1/d

foscavir 3/week

other or combination (specify)

54/ What is your first-line prophylactic approach for patients with ILD in the context of telomeropathy?

In R+ patients

valganciclovir

anti-CMV immunoglobulin

letermovir

other or combination

55/ What is your first-line prophylactic approach for patients with ILD in the context of telomeropathy?

In D+/R- patients

valganciclovir

anti-CMV immunoglobulin

letermovir

other or combination

56/ In what situations do you use anti-CMV immunoglobulin as an alternative to valganciclovir?

valganciclovir resistance

renal insufficiency

poor hematological tolerance

iterative replications

never or exceptionally

other

57/ In what situations do you use letermovir as an alternative to valganciclovir?

valganciclovir resistance

renal insufficiency

poor hematological tolerance

iterative replications

never or exceptionally

other

58/ in what situations do you accept a graft with a CMV mismatch (D+/R-)?

never

according to etiology (see question below)

according to degree of urgency

according to foreseeable difficulties of graft access (immunization size, blood group)

according to other criteria (please specify)

59/ For which etiologies do you never accept a graft with a CMV mismatch?

COPD

ILD without evidence of telomeropathy

ILD with confirmed or suspected telomere mutations

ILD associated with scleroderma

cystic fibrosis

pulmonary hypertension
